# The Influence of Radiation on Bone and Bone Cells—Differential Effects on Osteoclasts and Osteoblasts

**DOI:** 10.3390/ijms21176377

**Published:** 2020-09-02

**Authors:** Anna-Jasmina Donaubauer, Lisa Deloch, Ina Becker, Rainer Fietkau, Benjamin Frey, Udo S. Gaipl

**Affiliations:** Department of Radiation Oncology, Universitätsklinikum Erlangen, Friedrich-Alexander-Universität Erlangen-Nürnberg (FAU), 91054 Erlangen, Germany; Anna-Jasmina.Donaubauer@uk-erlangen.de (A.-J.D.); Lisa.Deloch@uk-erlangen.de (L.D.); Ina.Becker@uk-erlangen.de (I.B.); Rainer.Fietkau@uk-erlangen.de (R.F.); Benjamin.Frey@uk-erlangen.de (B.F.)

**Keywords:** osteoclast, osteoblast, bone, ionizing radiation, radiation therapy, bone microenvironment, osteocyte

## Abstract

The bone is a complex organ that is dependent on a tight regulation between bone formation by osteoblasts (OBs) and bone resorption by osteoclasts (OCs). These processes can be influenced by environmental factors such as ionizing radiation (IR). In cancer therapy, IR is applied in high doses, leading to detrimental effects on bone, whereas radiation therapy with low doses of IR is applied for chronic degenerative and inflammatory diseases, with a positive impact especially on bone homeostasis. Moreover, the effects of IR are of particular interest in space travel, as astronauts suffer from bone loss due to space radiation and microgravity. This review summarizes the current state of knowledge on the effects of IR on bone with a special focus on the influence on OCs and OBs, as these cells are essential in bone remodeling. In addition, the influence of IR on the bone microenvironment is discussed. In summary, the effects of IR on bone and bone remodeling cells strongly depend on the applied radiation dose, as differential results are provided from in vivo as well as in vitro studies with varying doses of IR. Furthermore, the isolated effects of IR on a single cell type are difficult to determine, as the bone cells and bone microenvironment are building a tightly regulated network, influencing on one another. Therefore, future research is necessary in order to elucidate the influence of different bone cells on the overall radiation-induced effects on bone.

## 1. Introduction

### 1.1. Osteoclast Differentiation and Function

The bone is a very dynamic tissue, undergoing extensive remodeling throughout the lifetime of an organism. In bone, a tight regulation between bone formation by osteoblasts (OBs), as well as bone resorption by osteoclasts (OCs) is necessary to maintain a functional skeletal system [1,2]. Bone metabolism is regulated by various hormones and cytokines and the dysregulation in this complex system can lead to numerous diseases characterized either by enhanced bone resorption (osteoporotic phenotype) or enhanced bone formation (osteopetrotic phenotype) [2]. 

The OC is a multinucleated giant cell, arising from the fusion of many mononuclear OC precursors with a myeloid/monocyte origin [3]. Size, as well as the number of nuclei, correlate positively with the bone-resorbing activities of OCs [4,5]. These highly specialized cells cannot only degrade the inorganic, mineralized components of the bone, which consist mainly of hydroxyapartite, but also the organic components of the matrix, which are composed mainly of collagen [6,7]. By adhering tightly to the bone surface via integrins, the OC generates a sealed resorption pit. This sealed zone is further supported by the reorganization of the actin cytoskeleton, in order to form an actin ring for an even stronger adhesion. In addition, the OC gets polarized and forms a ruffled border structure at the site of bone resorption. The fully differentiated OC secretes H^+^ through vacuolar ATPases into the resorption pit to resolve the inorganic components. Moreover, a variety of proteinases are secreted to dissociate extracellular matrix proteins. The enzymes for degradation are secreted via vesicle trafficking into the resorption pit, while degradation products of the bone matrix are finally endocytosed by the OC. Cathepsins (Cts) as well as matrixmetalloproteinases (MMPs) are the best characterized enzymes involved [7,8]. 

Analogous to the processes of bone remodeling and resorption that are tightly regulated, osteoclastogenesis from precursor cells is a strictly regulated differentiation process. Similar to all other hematopoietic lineage cells, OCs originate from hematopoietic stem cells (HSCs) in the bone marrow. More precisely, OC progenitors differentiate from the common myeloid progenitor lineage (CMP). Cells of this lineage give rise to monocytes and macrophages, dendritic cells and osteoclasts [9]. In the bone, the stimulation of the progenitor cells with the cytokines receptor activator of nuclear factor kappa B ligand (RANK-L) and macrophage colony-stimulating factor (M-CSF), amongst other factors, triggers a further differentiation into pre-osteoclasts. Here, M-CSF serves as a key survival signal, whereas RANK-L mainly induces OC-specific differentiation processes in progenitor cells [9,10]. In the bone, osteocytes, as well as OBs, but also immune cells, serve as a source of RANK-L, creating a local microenvironment that enables osteoclastogenesis specifically in bone tissue [11]. In the differentiating cell, these cytokine signals lead to an activation of nuclear factor kappa-light-chain-enhancer of activated B-cells (NF-ƙB), an essential transcription factor for osteoclastogenesis. NF-ƙB activation in turn, leads to an activation of c-fos and a nuclear factor of activated T cells 1 (NFATc1), the master transcription factor for osteoclastogenesis. Due to RANK-L stimulation, the expression of NFATc1 is strongly upregulated by autoamplification. Finally, OC-specific genes are induced, and amongst them are dendritic cell-specific transmembrane protein (*Dcstamp*), v-type proton ATPase subunit d2 (*Atp6v0d2*), tartate-resistant acid phosphatase (*Acp5*, TRAP) or Cathepsin K (*Ctsk*), enabling common OC functions [9]. By now, a mononuclear OC has formed.

For efficient bone resorption, the formation of multinucleated giant cells from the mononuclear osteoclast precursor cell is essential. For these differentiation processes, excessive arrangements occur in the mononuclear osteoclasts. Similar to the previously described differentiation processes, OC multinucleation is also a tightly regulated process. Amongst others, DC-STAMP and OC-STAMP (osteoclast stimulatory transmembrane protein) are essential fusogens in OC multinucleation. In summary, the multinucleation can be divided into three main steps. First, the cells need to come into close proximity by chemotaxis, adhere to one another, and finally become multinucleated cells by membrane fusion. The chemotactic approximation of the cells is mainly mediated through C–C motif chemokine ligand 2 (CCL2) signaling. Second, the approached cells subsequently adhere to one another by the expression of integrins and E-cadherin which initiates cytoskeletal rearrangements preparing the cellular fusion. Third, as the actin cytoskeleton reorganizes, membrane fusion is enabled leading to cell multinucleation. Finally, a fully functional, multinucleated OC has formed [12].

For the proper functionality of OCs, their tightly regulated interaction with bone-forming OBs is necessary. Differently from OCs, OBs arise from pluripotent mesenchymal stem cells (MSCs). The main function of OBs is the synthesis of new bone matrix. In detail, OBs secrete organic matrix components, such as collagens and proteoglycans to form the osteoid. Moreover, OBs secrete hydroxyapatite crystals via vesicles into the extracellular space, in order to build the inorganic component of the bone matrix. Amongst those functions, OBs also have a key role in the regulation of osteoclastogenesis. Here, they stimulate the differentiation of pre-OCs via the secretion of RANK-L, and MCS-F [13]. Bone, as a complex organ, contains numerous cell types, additionally to the bone-forming and resorbing cells. Next to OCs and OBs, osteocytes serve as a major component of bone. In this organ, osteocytes function as mechanosensory cells that pass their signals down to OBs and OCs in order to impact on bone homeostasis. Osteocytes develop from mature OBs that become embedded in the secreted bone matrix. These cells orchestrate bone resorption and formation, as the mechano-sensation leads to an adaption in the rate of bone formation and secretion. High mechanical loads trigger bone formation and reduce bone resorption, whereas mechanical unloading, e.g., in space travel, leads to the opposite effects. Appropriately, osteocytes can impact directly on OC, as well as on OB differentiation and activity [13,14].

Bone homeostasis, especially OC and OB differentiation and functionality are tightly regulated processes. Consequently, those processes are prone to numerous disruptive factors. Internal factors such as hormonal imbalances during menopause or rheumatic diseases can cause severe skeletal transformations [15,16]. On the other hand, also external influences can affect the bone in general, as well as OCs and OBs. Those factors include medical drugs, mechanical stress but also environmental or therapeutically delivered ionizing radiation (IR) [17,18,19,20].

### 1.2. Effects of Ionizing Radiation on Cells 

In general, there are numerous forms of radiation, as radiation is simply defined as a form of energy that spreads as an electromagnetic wave or particle radiation. The most relevant form of radiation for clinical applications and considerations is ionizing radiation (IR). In detail, IR can occur in various forms, such as alpha or gamma radiation or X-rays, which have distinct physical origins and properties. Thereby, also their biological effects on organisms and cells are various [21,22]. 

IR affects the molecules in cells when being absorbed by these. The absorbed energy induces the formation of free radicals in the cells, most commonly ROS (reactive oxygen species), which are highly reactive. IR damages different kinds of cellular molecules. Nonetheless, the destructive effects of IR on DNA molecules are the best characterized, as they lead to the most deleterious effects [23]. The damaging effects of IR on DNA range from simple base damages to complex double strand breaks or DNA crosslinks. These lesions induce several cellular repair mechanisms, such as base excision repair and homologous recombination. Nonetheless, these repair mechanisms fail, if the induced DNA damage is too extensive, finally leading to the induction of senescence, cell cycle arrest or cell death. However, if cells escape those mechanisms, mutated cells can be generated [23,24]. 

In addition to those targeted effects of IR, non-targeted effects, which are commonly immunological effects, can be initiated mainly through the immunogenic properties of the different forms of cell death. Necrotic cells for example, which are characterized by an uncontrolled cell lysis due to external factors, release damage associated molecular patterns (DAMPs) into the extracellular space, leading to the recruitment of immune cells and finally to their activation [24,25]. 

As all other tissues and cells, bone and the associated bone remodeling cells are also prone to radiation-induced damage. The effects of radiation on bone-associated cell types are especially important in questions of the medical application of IR, as well as for questions on environmental radiation effects [19,20,26].

This review article summarizes the current state of knowledge on the effects of IR on bone in general, but specifically on bone-resorbing OCs and bone-forming OBs. Here, we will focus especially on radiation in the therapeutic context, e.g., in cancer therapy or for the therapy of chronic degenerative and inflammatory diseases and on the effects of environmental radiation, e.g., in space travel. Moreover, the influence of IR on other cells of the bone microenvironment is discussed shortly.

At first sight, numerous studies discussed in this paper report results that seem contradictory in the context of the effects on bone and bone cells. Nonetheless, under the consideration of the radiation dose or the inflammatory status, the results discussed below fit into a common concept. Finally, perspectives of future research are reviewed in the outlook.

## 2. High Dose Radiation—IR in Cancer Therapy and the Influence on Bone Cells

As IR can induce cell death, radiation therapy (RT) is employed in cancer treatment for more than 50% of all cancer patients [27,28]. By irradiating tumors with fractionated doses of IR (single dose: ~2 Gray (Gy), total dose: 50–70 Gy), IR accumulates in the tumor tissue, leading to the death or senescence of fast dividing, malignant cells. Even though IR is delivered with high precision to the target tissue, the damage of adjacent healthy cells cannot be completely prevented [29].

As well, the bone is often affected by radiation damage. X-rays, as well as gamma rays, can change the primary structure of bone collagen, which is an essential part of bone, as it provides mechanical strength [30,31,32]. Consequently, many cancer patients undergoing RT suffer from side effects ranging from radiation osteitis to more severe complications such as fractures. Such complications have been described in numerous entities such as breast or brain cancer, or in pelvic tumor diseases [20,33,34,35]. Moreover, osteopenia or even osteoporosis are side effects that have been unavoidable after RT in distinct constellations [36]. 

Numerous studies suggest that bone damage is mainly due to IR-induced defects in bone-forming OBs. Therefore, the radiation effects on OBs are well studied, while the effects on OCs are less characterized. Since OBs are essential for a proper OC differentiation and thereby for a functional bone homeostasis, radiation effects on OBs will affect OC biology at least indirectly [8]. IR influences OB functionality, such as collagen production, but also OB proliferation. Furthermore, IR is reported to induce cell cycle arrest in OBs [37,38,39]. Some studies report that there is a reduced deposition of the bone matrix by OBs after the direct influence of IR on the bone, coming along with either stable or decreasing OB cell numbers in rodent studies [40,41,42,43]. In murine OB cell lines, X-rays provoked DNA double-strand breaks and consequently cell cycle arrest. These alterations led to a dose-dependent expression of transforming growth factor (TGF) -β1, which is known to modulate OB differentiation and mineralization [44]. Even though numerous studies report the deleterious effects of IR on OBs, some studies report a resistance of OBs to IR or even stimulating effects on these bone cells. Calvarial derived OBs, as well as OB-like MC3T3 cells, were proven to be relatively resistant to IR-induced apoptosis in a time- and dose-dependent manner [42]. When IR is applied in low doses in in vitro studies, it is even reported to promote OB proliferation and differentiation (see next section for further details) [45,46,47]. 

OBs influence OC differentiation by promoting OC progenitors with activation factors and cytokines [8,48]. Therefore, the aforementioned effects of IR on OBs will also indirectly affect OCs. As summarized above for OBs, the studies on IR effects on OCs also reveal conflicting results. Several published studies report either decreasing or stable OC numbers after RT [49,50,51,52,53]. Surprisingly, numerous reports even described increasing OC numbers following RT, suggesting that OCs may be key players in radiation-induced bone loss. Appropriately, the X-irradiation of mouse bones leads to increasing OC numbers with a higher activity [54]. In addition, even systemic bone loss can be induced in mice when only an isolated limb is exposed to high doses of IR [42]. In accordance with this, exposure to IR leads to an upregulation of the expression of OC marker genes in marrow tissue [55]. In addition to RT with X-rays, γ rays are also reported to lead to increasing OC levels in mice [56]. Yang et al. stated that γ rays may promote the differentiation of OC precursors while mature OCs are inhibited in their functions in murine RAW264.7 cells [57]. Especially when applied in low doses (under 0.5 Gy), γ rays may promote OC cell fusion coming along with the expression of OC marker genes, while doses higher than 0.5 Gy had the opposite effect in murine RAW264.7 cells [19]. In addition, X-rays, applied as a whole body irradiation of 2 Gy in mice, lead to an increase in osteoclastic differentiation and bone resorption, which is determined by increasing OC numbers and surface area, as well as increasing serum markers of bone resorption [58]. The differential effects of IR on OCs may depend on the applied dose of IR, as Zhang et al. found that low doses of X-rays promoted osteoclastogenesis, whereas high doses led to the reduced differentiation of OCs, probably by the induction of apoptosis and actin disorganization [26]. As high doses of IR induce a damage response and inflammatory processes in affected tissues such as bone, the secretion of pro-inflammatory cytokines, e.g., tumor necrosis factor (TNF) α, interleukin (IL) -6 and IL-1 may be stimulated. The differentiation of OCs in turn can be increased in such a rather pro-inflammatory milieu, as TNF-α and IL-1 directly stimulate the expression of RANK-L [54]. 

In summary, the deleterious effects of high doses of IR in cancer therapy are well described, whereas the underlying cellular mechanisms remain mostly elusive, as the effects on bone-forming cells are not well understood. This may be due to the various different factors in the studies mentioned above, that may influence the outcome, such as the radiation dose, the radiation source (heavy ions, γ rays or X-rays), as well as the cell type (murine, human) or the study design (in vivo or in vitro experiments), as well as the inflammatory status (as bone is tightly linked to the immune system).

## 3. Low-Dose Radiation—IR for Degenerative and Inflammatory Diseases and the Influence on Bone Cells

The therapeutic application of IR is not limited to cancer therapy. If delivered in low doses, IR does not induce cell death, senescence or inflammatory processes, but ameliorates pre-existing inflammation [59]. After the discovery of X-rays, they were rapidly applied for the treatment of rheumatoid arthritis and various diseases affecting the spine or joints [60,61]. Today, low doses of X-rays or α radiation are commonly applied for the therapy of chronic degenerative or inflammatory diseases, such as arthrosis or rheumatoid arthritis. Especially patients that are refractory for classical treatments and drugs, such as biologicals or non-steroidal anti-rheumatic drugs, can profit from exposure to low doses of radiation [62,63,64,65]. Progressive inflammatory processes and thus a painful destruction of cartilage and bone characterizes these diseases [66]. As an alternative treatment option, patients suffering from the aforementioned indications can receive low-dose radiation therapy (LD-RT). During therapy, patients are irradiated with fractionated low doses of X-rays with a single dose between 0.5 Gy and 1.0 Gy (total dose: 3–6 Gy). Furthermore, exposure to low doses of the radioactive noble gas radon can be applied as a spa therapy for the treatment of chronic degenerative and inflammatory diseases in order to induce pain-relieving and anti-inflammatory effects [62,63,67]. Even though exposure to low doses of IR can have analgesic and immunomodulatory effects, the underlying osteoimmunological mechanisms are not understood in detail. As bone destruction and remodeling are a hallmark of chronic degenerative and inflammatory diseases, the effects of low doses of IR on bone and bone-forming cells have already been examined in several studies and are still the subject of current research. 

The tight interaction of bone homeostasis and the immune system is well known (osteoimmunology). Low doses of IR are modulating the immune system by various molecular mechanisms [59,68,69] and thereby also impact on bone homeostasis. Amongst these mechanisms, the modulation of cytokine secretion by leukocytes and their adhesive behavior on endothelial cells, as well as the reduced production of nitric oxide (NO) by macrophages and granulocytes are well described [59]. Likewise, various studies performed in animal models of inflammation confirmed the positive impact of LD-RT [70,71,72,73,74]. For instance, LD-RT resulted in a less severe course of disease in arthritic mice with a reduction of the inflammatory area in the joints [70]. The positive impact of low doses of IR on chronic degenerative and inflammatory diseases, but especially on bone metabolism, is further supported by patient studies. In general, LD-RT or radon spa therapies are reported to lead to an amelioration of symptoms, for example in patients suffering from osteoarthritis or destructive processes of the spine and joints [63,75,76,77].

The effects of low doses of IR on cells of the bone, especially on OBs and OCs, has also been studied in more detail. As OBs produce bone matrix, they can potentially counteract the destructive processes in degenerative and inflammatory diseases. Therefore, OBs are a promising target in the therapy of chronic degenerative and inflammatory diseases. Appropriately, exposure to low doses of X-rays can have stimulatory effects on OBs. For instance, doses of 0.5 Gy of X-rays induce OB proliferation, as well as differentiation in murine calvaria-derived OBs [46]. Moreover, Xu et al. demonstrated that low doses of especially 0.5 and 1.0 Gy promote the differentiation and mineralization of murine OBs in vitro, but did not alter OB proliferation [45]. In addition to these findings at the cellular level, low doses of IR modulate OB biology also at the molecular level. For example, the expression of OB-specific genes, such as osteopontin and osteocalcin, were stimulated in murine calvarial-derived OBs after exposition to low doses of X-rays [47]. In accordance to these in vitro findings, results from animal studies verify the results described above. Even fracture healing can be promoted after the exposure to low doses of X-rays in rats [46]. Especially in the stadium of hard callus formation, low doses of X-rays promote the process of fracture healing [78]. In OBs from arthritic mice, irradiation with low doses of X-rays increases the mineralization rate in a dose-dependent manner, with 0.5 Gy being the most effective dose. This positive effect on the mineralization potential was even reproducible in OBs derived from mice of a non-inflamed background [70,79].

For OCs on the other hand, it is hypothesized that low doses of IR have a positive impact on bone homeostasis, as OCs are inhibited in their viability and their function. In accordance with this, numerous publications confirm the inhibitive effect of the low doses of IR on OC differentiation and functionality. Pramojanee et al. reported that X-rays in very low doses (about 1.5 mGy) reduce the production of ROS [80]. As ROS are required for the formation and activation of OCs [81,82], the reduction of cellular ROS by low doses of IR in OCs might inhibit osteoclastogenesis. Accordingly, in OCs derived from arthritic mice, LD-RT leads to reduced differentiation, along with a diminished bone-resorbing potential, with 0.5 and 1.0 Gy being the most effective doses [70]. As painful bone loss is a key characteristic of arthritis, LD-RT might counteract these processes in arthritis [70,83]. On the other hand, OCs derived from a non-inflamed, healthy background, showed a higher differentiation potential but a reduced bone-resorbing potential [79]. These findings indicate that the effects of low doses of IR on bone cells might also be dependent on the inflammatory state. Patient studies confirm the positive effect of low doses of IR on the bone metabolism. Likewise, radon spa treatments lead to a reduction of serum markers of bone resorption in patients [84], confirming the previously discussed preclinical findings. In summary, low doses of IR have positive effects on the bone homeostasis, especially in inflammatory settings. OBs, as well as OCs, are modulated leading to promoted bone formation and reduced bone resorption. Nevertheless, the applied dosage, the radiation source and quality, as well as the inflammatory state have an impact on the outcome and should therefore be carefully taken into consideration when treating patients.

**Figure 1:**
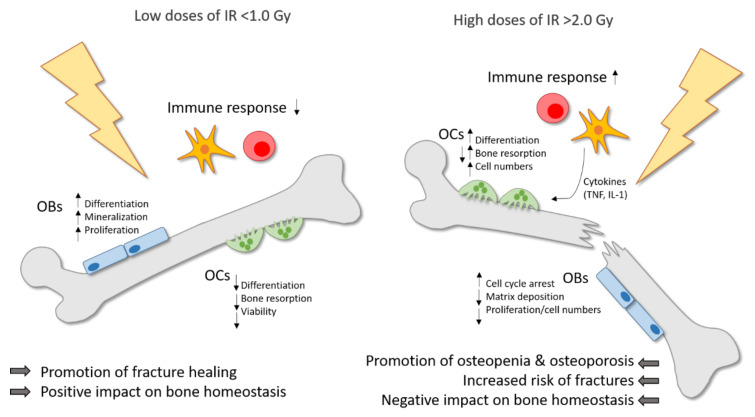
Ionizing radiation (IR) induces differential effects on bone in dependence of the dose. Low doses of IR (≤1.0 Gy) are commonly applied for the therapy of chronic degenerative and inflammatory diseases and can counteract the destructive processes on bone with a positive impact on bone homeostasis and a promotion of fracture healing. Pre-existing inflammatory processes are downregulated by low doses of IR. Moreover, the differentiation, mineralization and proliferation of osteoblasts (OBs) is stimulated. On the contrary, the differentiation, viability and bone-resorbing capacity of osteoclasts (OCs) is reduced. In high doses (≥2.0 Gy), IR is usually applied in cancer therapy and has a negative impact on bone homeostasis with a higher risk of osteopenia and osteoporosis, and finally an increased fracture risk. In OCs, the differentiation, bone resorption and cell numbers are enhanced, whereas in OBs, the matrix deposition and proliferation rate are reduced and cell cycle arrest is induced. In addition, high doses of IR induce the secretion of pro-inflammatory cytokines by immune cells, which in turn can stimulate OCs.

When comparing the effects of high doses of IR on bone and bone-remodeling cells, the effects of low doses of IR often seem to induce contrary effects. Therefore, the influence of low doses of IR on bone and in general on inflammatory and degenerative diseases is still subject to current research. In summary, one can conclude that high doses of IR lead to bone destruction with increased bone resorption by OCs and reduced bone formation by OBs, while on the other hand, IR applied in low doses has a positive impact on bone homeostasis, with an increased mineralization rate and reduced bone resorption. Figure 1 summarizes these findings.

## 4. IR in Space Travel and the Influence on Bone Cells

Low doses of IR do not only play a role in chronic degenerative diseases and in medical diagnostic procedures, but also in the exposure to environmental radiation. Cosmic radiation accounts for a considerable amount of the yearly radiation dose a human is exposed to. The effects of cosmic radiation on the human body are even more pronounced and important in space travel, as astronauts are exposed to high doses of high energy radiation. These doses range between approximately 0.4 up to 0.8 mGy/day during extended space flights [85]. In comparison to the radiation source applied for medical applications (mainly X-rays), cosmic radiation consists of different kinds of radiation. In detail, space radiation consists of heavy charged particles of a high linear energy transfer (LET), as well as mainly of high energy protons [86]. X-rays and γ-rays on the other hand, are considered as low LET electromagnetic rays. Therefore, a direct comparison between the low LET IR that is commonly used for medical applications and the high LET IR that astronauts are exposed to in space is not feasible, as high and low LET have different physical and biological properties [87,88]. Cosmic radiation has numerous negative effects on the human body during space travel, with the most prominent ones being a significant increase in the individual cancer risk, cardiovascular deconditioning, changes in the immune system and also alterations in bone homeostasis, and consequently in a loss of bone mass [89,90]. In addition, microgravity in space further promotes bone loss, making the space environment especially deleterious for the skeletal system and bone [91]. Therefore, effort has been applied into the examination of the effects of radiation on the human body in general, and particularly on bone, in the context of space travel.

It is well known that the application of high LET IR in space travel-relevant doses can induce bone loss. The loss of skeletal mass is due to an increased bone resorption activity, which is accompanied by an inhibition of bone formation [56,92]. Several studies on animal models have proven that the application of space travel-relevant doses induces bone loss. Likewise, whole-body exposure to 2 Gy of γ rays, protons or ions that should mimic the mixed IR exposure during a space mission, induces a reduction of the quantity and quality in trabecular bones [93]. Accordingly, the exposure to low doses of protons (1 Gy) or heavy ions (<0.5 Gy) that resemble the space environment, can induce bone loss lasting for several weeks up to months [94,95]. 

The bone loss described during space travel might be primarily due to the effects of IR on OCs. The majority of the publications on the effects of space IR focus on the effects on OC biology, whereas the effects on OBs are scarce. Likewise, rising OC numbers and the elevated levels of serum markers of bone resorption were also reported after a whole-body exposure of mice to IR [96]. Moreover, also γ rays in space travel-relevant doses negatively affect bone architecture and increase OC numbers in mice [56]. Studies on OB biology in the context of space radiation, on the other hand, are scarce. Yumoto et al. reported that irradiation with low doses of iron ions inhibited the growth of murine bone marrow cells under osteogenic conditions, indicating that osteoblastic development is impaired following RT [97]. Nevertheless, most studies examined the effects of IR on osteoblasts in the context of high-dose IR in the context of cancer therapy (see 2. High dose radiation—IR in cancer therapy and the influence on bone cells). The mechanisms reported in this context might also play a role during exposure to cosmic radiation, even though they are not directly applicable to space travel, as space radiation consists mainly of high LET IR that has different physical properties. The combined effects of cosmic radiation and microgravity in space have been reported to synergistically act on the phenomenon of bone loss during space travel [19,97]. Exposure to microgravity alone is reported to promote OC activities in vivo and enhance OC differentiation in vitro [98,99]. In addition, microgravity increases cortisol levels in OB cultures in space, which is a well known inhibitor of OB differentiation [100]. In mice, the combination of musculoskeletal disuse and exposure to γ rays causes acute cancellous bone loss, by increasing bone resorption by OCs [101]. In addition, low doses of γ rays promote OC cell fusion, whereas the addition of microgravity induces even higher levels of cell fusion. Interestingly, these two environmental factors act as additives on OC cell fusion. In accordance, partial weightbearing and exposure to low doses of high-LET radiation negatively affected the maintenance of bone mass in mice due to higher levels of bone resorption and reduced bone-forming capacities [102]. 

These results on the effects of space IR on bone seem contradictory at first sight, as low doses of IR are also used to treat bone loss in chronic degenerative diseases. In space travel, on the other hand, low doses of IR lead to bone loss. In the context of space travel, astronauts are exposed to mixed beams over a long period of time that act synergistically with microgravity on bone. Moreover, space radiation mainly consists of high LET IR, whereas radiation in the therapeutical context is mainly low LET IR. As space travel will become an even more important topic in the future, further research in this field is necessary. The effects of low doses of space radiation on OBs especially remain mostly elusive.

## 5. The Influence of IR on the Bone Microenvironment

The previous sections of this review mainly focused on the influence of IR on bone in general with a special focus on bone-resorbing OCs and bone-forming OBs. As bone is a complex organ, many other cell types in the bone microenvironment may be affected by IR and influence bone homeostasis. In addition to OBs and OCs, osteocytes also have a key role in bone homeostasis. In bone, osteocytes are the most abundant cell type and have a key role in the regulation of bone metabolism. Osteocytes reside in lacunae and communicate with one another and other cell types of the bone, such as OBs or marrow cells [103]. The effect of IR on osteocytes has not been examined in detail to date. In vitro experiments indicate that IR induces morphological changes in cultured osteocytes, as they show a polygonal shape with shortened dendrites after exposure to IR in a dose range that resembles the cancer treatment situation. Furthermore, IR reduces cellular viability in osteocytes and increases the apoptosis rate in these cells. Interestingly, irradiated osteocytes can promote osteoclastogenesis through the release of HMGB1 (high mobility group box 1) followed by an elevation of the RANK-L/OPG (osteoprotegerin) levels, indicating a key role for osteocytes in IR-induced bone loss during cancer therapy [104]. Moreover, in vivo studies have proven that very high doses of X-rays (30 Gy) lead to significant changes in the osteocyte lacunae network, with a decrease in osteocytes in woven bone, accompanied by increased numbers of empty lacunae [105]. These studies indicate that osteocytes may have a key role in IR-induced bone loss, as osteocytes are key regulators of the bone mineral metabolism, influencing OB and OC activity [106].

On the other hand, radiation also affects bone vasculature. The vascular system has an essential role in bone homeostasis, as the transport of nutrients and oxygen depends on vascularization and additionally, the vasculature enables the communication between endothelial cells and osteoclasts. Thereby, the vascular system influences bone remodeling [107]. As early as 1926, Ewing reported that radiation affects bone vasculature, and that these changes in the haversian channels lead to the formation of sclerotic connective tissue in the marrow [108]. Since then, several studies reported the deleterious effects of IR on bone vasculature. In vivo studies in mice and rats demonstrated that irradiation with varying doses leads to a reduced blood flow in the irradiated bone [109,110]. Moreover, the bone destruction in rat calvaria after exposure to IR can be reversed by the induction of vascularization and by the local application of vascular endothelial growth factor (VEGF), demonstrating that the destruction of bone vasculature was closely linked to general radiation-induced defects in bone [111]. Further animal studies confirmed that IR leads to a reduced number of vessels and an increased distance between vessels in bone coming along with radiation-induced osteopenia [112].

IR in high doses is known to induce inflammation, along with an enhanced expression of pro-inflammatory cytokines in the irradiated tissue [113,114]. Pro-inflammatory cytokines in turn, such as IL-6 and TNF, are known to induce osteoclastogenesis, especially in the presence of RANK-L [115,116,117,118]. In accordance, it is reported that high doses of IR lead to enhanced osteoclastogenesis along with increasing levels of pro-inflammatory cytokines [119]. Low doses of IR, on the other hand, which are known to have rather anti-inflammatory effects, are reported to reduce OC differentiation and functionality [70].

The bone marrow is a further important part of the bone microenvironment, as it gives rise to hematopoietic, as well as mesenchymal stem cells, which in turn can differentiate into OCs and OBs, respectively [120]. As mentioned earlier, osteoclasts develop from the myeloid lineage of HSCs from the bone marrow. For the proper differentiation of OCs, the local microenvironment is of great importance, as the surrounding cells of the bone secrete RANK-L and M-CSF amongst other factors that trigger osteoclastogenesis [9]. OBs on the other hand, differentiate from MSCs into pre-osteoblasts that migrate to the site of bone resorption, where they differentiate into fully functioning OBs that deposit the new bone matrix [121]. As OBs can differentiate further into osteocytes, MSCs consequently also give rise to these cells [120]. Additionally, OBs and OCs directly impact the differentiation of one another from the aforementioned precursor cells. Therefore, radiation damage to the bone marrow stem cells (MSCs and HSCs) also impacts bone homeostasis. In the case of MSCs, which give rise to OB progenitors, X-rays are either reported to stimulate proliferation at very low doses [122], or reduce the numbers of MSCs when irradiated with higher doses of X-rays, as they are usually applied during cancer therapy. These high doses also impact on the osteogenic potential of MSCs [123]. Chronic exposure to IR (applied as a Strontium radionuclide) also leads to a reduction of the proliferative capacity of MSCs, as well as their capability to support hematopoietic processes in a dose-dependent fashion [124]. Thereby, the effects of IR on MSCs can directly impact bone homeostasis. For HSCs, the impact of IR reveals conflicting results. On the one hand, IR is reported to cause apoptosis in hematopoietic stem cells, when they are exposed to the low doses of high energy and high charge radiation that are relevant in space travel [125]. On the other hand, Li et al. reported that very low doses of X-rays act as stimulants of hematopoietic progenitor cells, as their proliferation and mobilization was increased in an animal study [126]. These differential effects might be due to different radiation doses or radiation quality, as IR is also reported to have differential effects on bone remodeling (see the above sections of this review).

**Figure 2:**
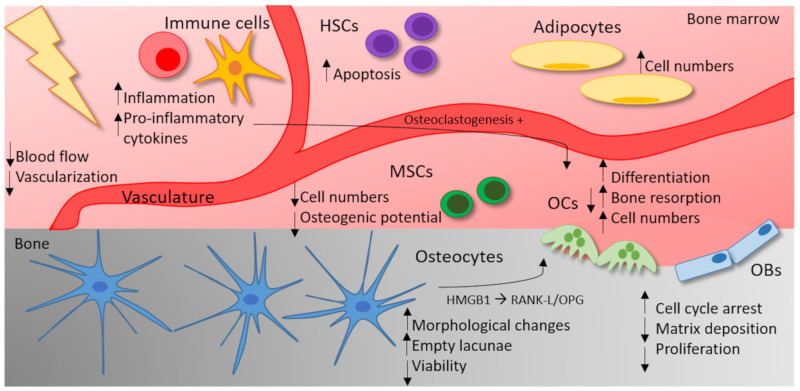
The influence of high doses of IR on the bone microenvironment. High doses of IR (≥2 Gy) impact on different cell types of the bone microenvironment. In OCs, IR increases the differentiation, the bone-resorbing capacity and the cell numbers. In OBs, on the other hand, IR leads to cell cycle arrest and a reduced matrix deposition and proliferation rate. Osteocytes react with morphological changes, a reduced viability and therefore empty lacunae. By the secretion of HMGB1 and an increase in the RANK-L/OPG (osteoprotegerin) levels, irradiated osteocytes trigger OC differentiation. In the bone marrow, IR also affects the vasculature, resulting in a reduced blood flow and vascularization. Moreover, IR leads to an increased marrow adiposity. The apoptosis rate of hematopoietic stem cells (HSCs) increases after exposure to high doses of IR. In mesenchymal stem cells (MSCs), high doses of IR are either reported to stimulate proliferation or reduce cell numbers in a dose-dependent fashion. In addition, the osteogenic potential of MSCs can be reduced by high doses of IR. IR can trigger an inflammatory response and the secretion of pro-inflammatory cytokines (TNF, IL-1) can stimulate osteoclastogenesis.

In addition to the direct effects on MSCs and HSCs, IR leads to an immune-mediated cytokine response in bone marrow [96,127,128]. Exposure to γ radiation or heavy-ion radiation is reported to increase the gene expression of pro-osteoclastogenic genes such as *Rankl*, not only in mineralized, but also in marrow tissue. In addition, IR also induces the expression of pro-inflammatory and pro-osteoclastogenic factors, such as TNF, Il-6 and MCP1 in the marrow tissue of irradiated mice. Thereby, IR leads to an accumulation of OC precursors in the marrow and acts by stimulating developing as well as mature OCs [55]. Additionally, high doses of X-rays are reported to induce marrow adiposity, which means that the number of adipocytes increases in the bone marrow after irradiation [129]. As the bone marrow gives rise to OB and OC progenitors [130], marrow adiposity could negatively impact on the differentiation of bone remodeling cells. Likewise, in cancer patients, the reduction of the bone volume after radiation therapy comes along with an increase in marrow adiposity [131,132]. As the effects of IR on the bone microenvironment are complex, the current state of knowledge on the influence of high doses of IR is summarized in Figure 2.

In summary, the bone microenvironment consists not only of OCs and OBs, but also of various cell types that influence bone remodeling. The previous section demonstrated that IR-mediated effects on bone are not only due to changes in OB and OC biology, rather than being dependent on changes in the whole bone microenvironment. Nonetheless, the effects are complex, as the bone cells influence one another. This is why further research is necessary to understand the effects of IR on the bone microenvironment in detail.

## 6. Conclusion and Outlook

In this paper, the effects of IR on bone and bone remodeling cells is discussed with a special focus on bone-resorbing OCs and bone-forming OBs. In summary, the presented in vivo and in vitro studies suggest that the effects of clinically utilized IR on OBs and OCs are mainly dependent on the applied radiation dose. If applied in high doses, IR is reported to primarily have deleterious effects on bone with an increase in bone resorption and a decrease in bone formation. Low doses of IR, on the other hand, induce contrary effects: as in the context of chronic degenerative and inflammatory diseases, IR in low doses downregulates the differentiation and functionality of OCs, whereas OBs are stimulated. 

Nonetheless, numerous factors might additionally account for the different results achieved in the studies. The findings on the effects of IR on bone and bone cells come from in vitro and in vivo (mainly animal) studies. As in vivo studies can focus on bone as a whole organ, in vitro studies mostly present an isolated view on a particular cell type. Therefore, the experiments with cultured cells have the opportunity to examine the response of a cell type in more detail, whereas in vivo studies can provide more information on the interplay of the different cells of the bone microenvironment. Different results obtained in the studies can be explained by the fact that IR effects on isolated cell types might differ from the in vivo situation as different bone remodeling cells impact on one another. Furthermore, in the intact bone environment, immune-mediated inflammatory processes can additionally act on the bone. These inflammatory mechanisms and the whole bone microenvironment cannot be simulated in cell culture systems. 

Not only are the cell types differentially affected by IR, the developmental status of the cells might also be a key factor, as it is well known that the stage of differentiation impacts on radiation susceptibility and the cellular radiation response [46]. Likewise, the radiation response of bone stem cells differs from the response of mature bone cells. 

In addition to the different radiation doses applied in the studies, the radiation source and quality can also affect the outcome. In the field of the therapeutical application of IR, X-rays are the major radiation source, whereas in the context of space travel, IR is a mixture of different sources, such as protons, X-rays and heavy charged particles [86]. Different kinds of IR are well known have distinct physical properties that can result in differential biological effects [22]. 

In summary, the here presented studies provide an overview of the IR-induced effects on bone, indicating that especially the radiation dose and the bone microenvironment have an impact on the outcome. Nonetheless, further research is necessary in order to harmonize the different findings on the effects of IR on bone.

Most studies are rather descriptive and focus mainly on determining the effects of IR on bone remodeling by quantifying the number of bone-forming and -resorbing cells, by analyzing the expression of marker genes or examining the bone-resorbing and mineralization capacities. Moreover, the studies discussed above are rather difficult to compare, as the scientific question as well as the set-ups vary extremely. For instance, different radiation sources such as protons, X-rays or γ-rays or local irradiation and whole-body irradiation cannot be compared directly. Therefore, future research should focus on analyzing the molecular mechanisms that lead to the radiation-induced effects on bone. Here, the cellular and molecular mechanisms in OCs can be of special interest. In OCs, especially the bone-resorbing capacities and the development are affected by IR. An important step in OC development is OC multinucleation, which is required for a proper bone-resorbing activity, as the number of nuclei correlates positively with the bone resorption efficiency. Furthermore, multinucleation requires multiple cellular components, such as the actin cytoskeleton, the membrane, cell adhesion molecules and the cell cycle machinery [12]. All of these cellular components might be affected by IR, leading to impaired multinucleation. Therefore, OC multinucleation might be a promising target for unraveling the cellular mechanisms that are directly or indirectly targeted by IR.

## Abbreviations

OC	Osteoclast
OB	Osteoblast
Cts	Cathepsin
MMP	Matrixmetalloproteinase
HSC	Hematopoietic stem cell
CMP	Common myeloid progenitor
RANK-L	Receptor activator of nuclear factor kappa B ligand
NF-ƙB	Nuclear factor kappa-light-chain-enhancer of activated B-cells
NFATc1	Nuclear factor of activated T cells 1
M-CSF	Macrophage colony-stimulating factor
DC-STAMP	Dendritic cell-specific transmembrane protein
TRAP	Tartrate-resistant acid phosphatase
CtsK	Cathepsin K
OC-STAMP	Osteoclast stimulatory transmembrane protein
CCL2	C-C motif chemokine ligand 2
IR	Ionizing radiation
ROS	Reactive oxygen species
DAMPs	Damage-associated molecular patterns
RT	Radiotherapy
Gy	Gray
TGF-β	Transforming growth factor β
TNF-α	Tumor necrosis factor α
IL	Interleukin
LD-RT	Low-dose radiation therapy
NO	Nitric oxide
LET	Linear energy transfer
OPG	Osteoprotegerin
HMGB1	High mobility group box 1
VEGF	Vascular endothelial growth factor
